# NSUN2 promotes colorectal cancer progression by enhancing SKIL mRNA stabilization

**DOI:** 10.1002/ctm2.1621

**Published:** 2024-03-11

**Authors:** Shaomin Zou, Yizhi Huang, Ziqing Yang, Jieping Zhang, Manqi Meng, Yijing Zhang, Junyan Feng, Rui Sun, Weiyao Li, Wencong Wang, Jesús García‐Foncillas López, Lekun Fang

**Affiliations:** ^1^ Department of General Surgery The Sixth Affiliated Hospital Sun Yat‐sen University Guangzhou China; ^2^ Guangdong Provincial Key Laboratory of Colorectal and Pelvic Floor Disease The Sixth Affiliated Hospital Sun Yat‐sen University Guangzhou China; ^3^ Biomedical Innovation Center The Sixth Affiliated Hospital Sun Yat‐sen University Guangzhou China; ^4^ Department of Biomedical Sciences City University of Hong Kong Hong Kong China; ^5^ Jiménez Díaz Foundation University Hospital Madrid Spain

**Keywords:** 5‐methylcytosine modification, colorectal cancer, NSUN2, SKIL

## Abstract

**Background:**

NOP2/Sun domain 2 (NSUN2) is one of the important RNA methyltransferases catalyzing 5‐methylcytosine (m5C) formation and participates in many critical bioprocesses. However, the roles and underlying molecular mechanisms of NSUN2‐mediated m5C modification in colorectal cancer (CRC) remain unclear.

**Methods:**

To explore the NSUN2 expression in CRC, fresh tissue samples were collected and Nsun2 knockout mouse was constructed. In vitro and in vivo functional assays were conducted to assess the role of NSUN2. RNA array and bisulfite sequencing were used to investigate the potential targets. The mechanisms of NSUN2 function on SKIL were identified by m5C‐methylated‐RNA immunoprecipitation and RNA stability assays. Additionally, tissue microarray analysis was conducted and patient‐derived tumour xenograft mouse (PDX) models were used to define the potential therapeutic targets.

**Results:**

NSUN2 was highly expressed in CRC and correlated with poor CRC patient survival. Moreover, silencing NSUN2 suppressed CRC tumourigenesis and progression in Nsun2 knockout mouse models. In vitro and in vivo studies suggested that NSUN2 promoted colorectal cancer cell growth. Mechanistically, SKI‐like proto‐oncogene (SKIL) is positively regulated by NSUN2, and the NSUN2‐SKIL axis is clinically relevant to CRC. NSUN2 induced m5C modification of SKIL and stabilized its mRNA, which was mediated by Y‐box binding protein 1 (YBX1). Elevated SKIL levels increased transcriptional coactivator with PDZ‐binding motif (TAZ) activation.

**Conclusions:**

Our findings highlight the importance of NSUN2 in the initiation and progression of CRC via m5C‐YBX1‐dependent stabilization of the SKIL transcript, providing a promising targeted therapeutic strategy for CRC.

## Graphical abstract

1. NSUN2 is highly expressed in colorectal cancer.
2. NSUN2 induces m5C modification of SKIL and promotes the stability of SKIL mRNA.
3. YBX1 might be the m5C reader of SKIL.
4. NSUN2 activates TAZ expression by SKIL.

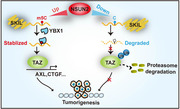

## 1 INTRODUCTION

Colorectal cancer (CRC) is the third most common cancer worldwide.^1^, ^2^, ^3^, ^4^ Surgery is the cornerstone of curative treatment. Nevertheless, most CRC patients succumb to the disease as a result of late diagnosis and ineffective treatment modalities.^5^, ^6^, ^7^ Therefore, novel treatment targets for CRC are urgently needed.

Recent discoveries have shown that aberrations in epigenetic regulation, such as RNA methylation, play crucial roles in cellular functions and numerous human diseases.^8^, ^9^ Recently, 5‐methylcytosine (m^5^C) modification has attracted increasing attention.^10^, ^11^ In eukaryotes, the deposition of m^5^C on mRNA is mostly catalyzed by the NOP2/Sun domain (NSUN) family^12^, ^13^, ^14^, ^15^ and tRNA aspartic acid methyltransferase 1 (TRDMT1) is found to mainly methylate tRNA.^16^, ^17^ NSUN2, also called myc‐induced SUN domain‐containing protein, participates in tumourigenesis and developmental disorders in a m^5^C‐dependent manner.^18^, ^19^, ^20^ Small ubiquitin‐like modifier −2/3 can interact directly with NSUN2 by stabilizing it and mediating its nuclear transport.^21^ NSUN2‐mediated methylation of the p16 3′UTR reduces p16 mRNA decay.^22^ However, its regulatory mechanisms and role in human CRC remain largely unknown.

SKIL, also known as SnoN, is a negative controller of transforming growth factor‐beta (TGF‐β) signalling.^23^, ^24^, ^25^ In mammals, SnoN is ubiquitously expressed in embryos and adult tissues.^26^ SKIL expression levels are elevated in many cancer cells and tissues, including those derived from non‐small‐cell lung cancer (NSCLC)^27^ and breast cancer.^26^ Evidence has shown that SnoN interacts with multiple components of the Hippo pathway, inhibiting the binding of large tumour suppressor kinase 2 (Lats2) to TAZ and leading to TAZ stabilization.^26^ Additionally, SKIL promoted tumourigenesis and immune escape of NSCLC cells through the upregulation of the TAZ/autophagy axis and inhibition of the STING pathway.^27^ In contrast, some studies have suggested that SKIL plays a tumour suppressor role.^25^, ^28^ For example, silencing SKIL increased the proliferation of ESCC cell lines, demonstrating the complex roles of SKIL in tumourigenesis.

Herein, we used NSUN2 knockout mice to uncover the oncogenic role of NSUN2‐mediated RNA m^5^C modification in CRC. Methylation by NSUN2 promotes the stability of SKIL mRNA in a Y‐box binding protein 1 (YBX1)‐dependent manner, ultimately upregulating TAZ expression. We propose that the NSUN2‐m^5^C‐SKIL‐TAZ axis promotes the initiation and progression of CRC.

## 2 RESULTS

### 2.1 Overexpression of NSUN2 in CRC tissues is associated with patient survival and tumourigenesis

**FIGURE 1:**
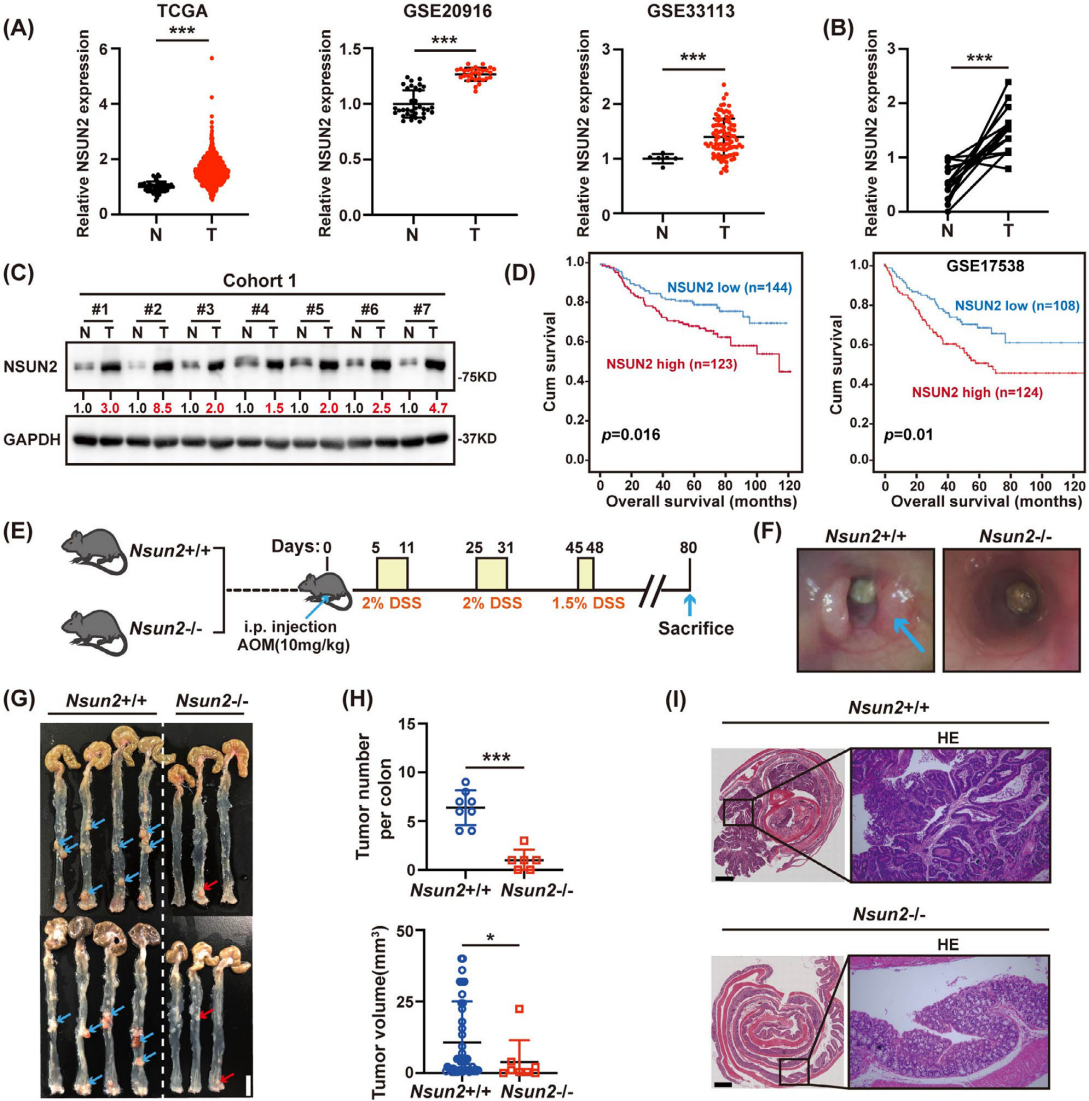
NSUN2 is upregulated in CRC, associated with patient's survival and tumourigenesis. (A) NSUN2 was highly expressed in colorectal cancer tumour tissues (T) compared with adjacent normal tissues (N) from TCGA, GSE20916 and GSE33113 databases. (B) qRT‐PCR analysis showed the NSUN2 mRNA levels in 14 paired samples of CRC tumours (T) and corresponding adjacent normal tissues (N). (C) Western blot analysis showed the NSUN2 protein levels in paired samples of CRC tumours (T) and corresponding adjacent normal tissues (N). (D) Kaplan–Meier estimates of survival time of patients in cohort (*n* = 267) from the sixth Affiliated Hospital of Sun Yat‐sen University and GSE17538 (*n* = 232) by different NSUN2 expression levels in tumours. (E) Schematic representation of azoxymethane (AOM)/dextran sulfate sodium (DSS). (F) Colonoscopy photos of representative examples from *Nsun2*+/+ and *Nsun2*‐/‐ mice. Arrow points to tumours. (G) Representative colon tumour images of *Nsun2*+/+ and *Nsun2*‐/‐ mice. Arrow points to tumours. Scale bar = 1 cm. (H) Tumour numbers and volumes of *Nsun2*+/+ and *Nsun2*‐/‐ mice. (I) H&E (hematoxylin and eosin) staining of colon tumour sections from *Nsun2*+/+ and *Nsun2*‐/‐ mice. Scale bar = 1 mm. *P < 0.05; **P < 0.01; ***P < 0.001.

To explore the role of mRNA m^5^C modification in CRC, we evaluated the expression levels of major mRNA m^5^C methyltransferases in a CRC cohort from The Cancer Genome Atlas database (TCGA) (Figure 1A and Figure S1A). There were no obvious differences in NSUN6, NSUN7 and TRDMT1 expression between the colorectal tumour tissues and adjacent normal tissues. Interestingly, the expression level of NSUN2 was higher in tumour vs. matched normal tissues. These findings were also verified in different CRC datasets, including GSE20916, GSE33113 and GSE8671 (Figure 1A and Figure S1B). Furthermore, the overexpression of NSUN2 in CRC tissues was also found in freshly collected tissues (Figure 1B, C and Figure S1C). Kaplan–Meier survival analysis showed that CRC patients with high NSUN2 expression had a lower overall survival (OS) than those with low NSUN2 expression (Figure 1D and Table S1). These results suggest that NSUN2 is frequently upregulated in CRC patients and might serve as a prognostic indicator for CRC.

We next generated NSUN2 knockout mice (*Nsun2*‐/‐) to investigate the role of NSUN2 in CRC tumourigenesis. Compared with those of *Nsun2*+/+ littermates, colon extracts of *Nsun2*‐/‐ mice showed a significant reduction in NSUN2 protein levels (Figure S1D). Then, a single intraperitoneal dose (10 mg/kg of body weight) of azoxymethane (AOM) followed by three six‐day cycles of oral administration of 2% or 1.5% dextran sulfate sodium (DSS) was administered to establish a CRC model (Figure 1E). As expected, *Nsun2*‐/‐ mice developed smaller and fewer tumours than *Nsun2*+/+ mice (Figure 1F–H). Histopathological analysis showed advanced colorectal tumour stages in *Nsun2*+/+ mice compared with *Nsun2*‐/‐ mice (Figure 1I), indicating the critical role of NSUN2 in the tumourigenicity and progression of CRC.

### 2.2 NSUN2 enhances the tumourigenesis and progression of CRC

**FIGURE 2:**
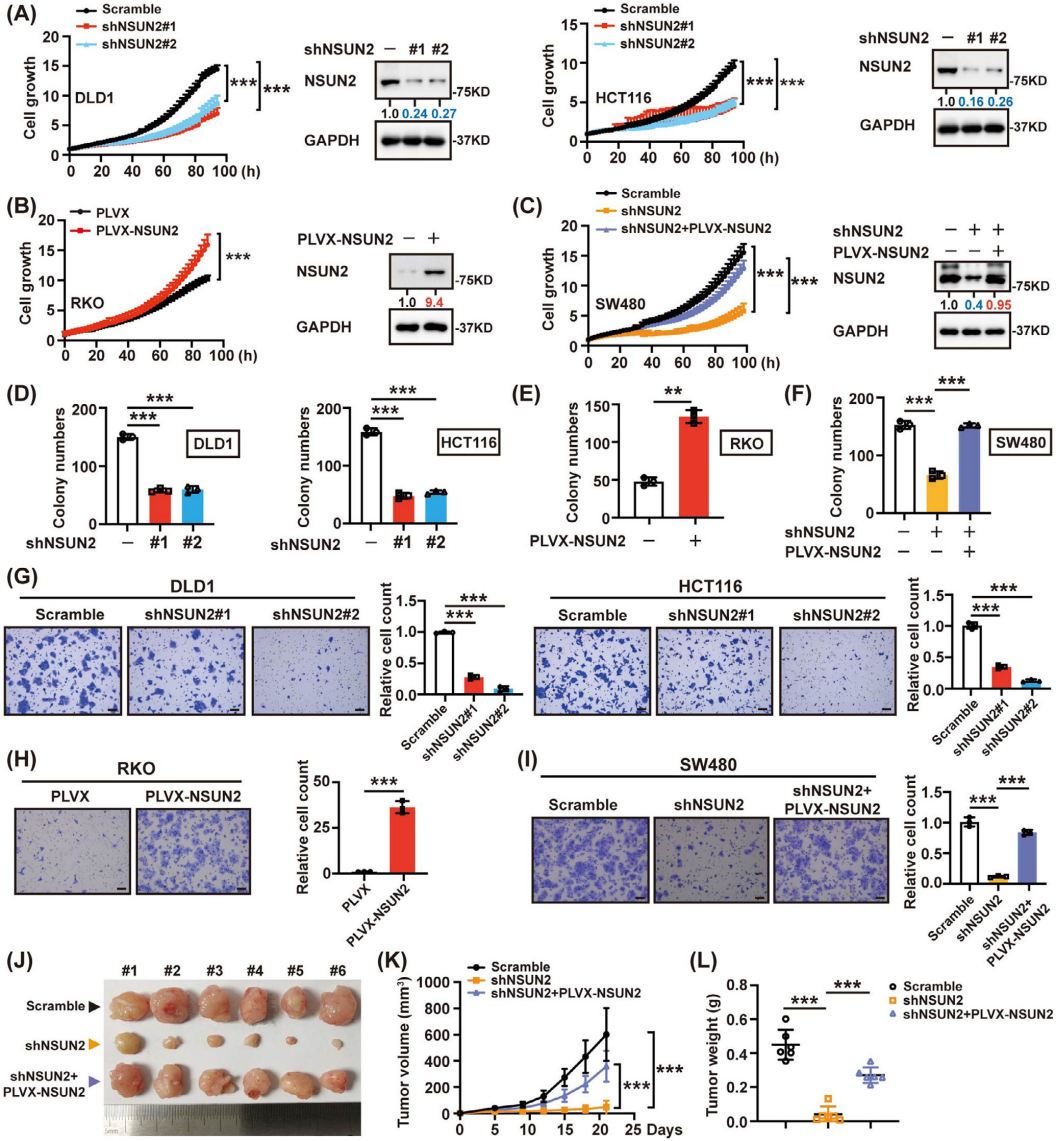
NSUN2 promotes malignant phenotypes of CRC cells both in vitro and in vivo. (A) After infection of DLD1 and HCT116 cells with shRNAs, cell growth curves were determined by Incucyte assays. The expression of NSUN2 was analyzed by Western blot. (B) Overexpression NSUN2 promoted CRC cell growth determined by Incucyte assays. (C) Overexpression of NSUN2 restored the cell growth inhibition caused by NSUN2 depletion. (D) Effects of NSUN2 depletion on the clonogenic ability of DLD1 and HCT116 cells. (E) Overexpression of NSUN2 promoted CRC cell colony formation. (F) Overexpression NSUN2 restored the reduced colony numbers caused by NSUN2 silencing. (G) After infection of DLD1 and HCT116 cells with shRNAs, cell migration was determined by transwell assays. Scale bar = 100 µm. (H) Overexpression of NSUN2 promoted CRC cell migration. (I) Overexpression NSUN2 restored the cell‐migration inhibition caused by NSUN2 silencing. (J) A xenograft mouse model was constructed by subcutaneously injecting nude mice with DLD1 cells (*n* = 6 per group). Representative images of xenografts were shown. (K, L) Decreased tumour volume (K) and weight (L) were observed in the NSUN2 knockdown group, which was restored by NSUN2 overexpression. **P < 0.01; ***P < 0.001.

To investigate the effects of NSUN2 on the behaviour of CRC cells, we knocked down *NSUN2* with shRNAs and determined the efficiency by western blotting. Incucyte and colony formation assays were used to explore the regulatory effect of NSUN2 on the proliferation of CRC cells. As Figure 2A, B shows that depletion of NSUN2 inhibited cell proliferation while overexpressing NSUN2 showed the opposite effects. In addition, reintroducing NSUN2 rescued CRC cell growth (Figure 2C). Consistently, colony formation assays showed that NSUN2 promoted colony formation ability, as indicated by colony numbers (Figure 2D–F and Figure S2A–C). We also found that the number of migrating cells significantly decreased after NSUN2 knockdown (Figure 2G). In contrast, upregulation of NSUN2 increased cell migration ability (Figure 2H). Rescue experiments suggested that NSUN2 was involved in promoting the migration of CRC cells, further confirming the oncogenic role of NSUN2 (Figure 2I).

Stably infected DLD1 cells were inoculated into the flanks of nude mice, and the effect of NSUN2 on xenograft tumour growth was observed. Compared with the control group, the NSUN2 knockdown group showed significantly decreased tumour sizes and weights, which could be rescued by reintroducing NSUN2 (Figure 2J–L). Collectively, these results indicate that NSUN2 promotes CRC growth both in vitro and in vivo.

### 2.3 NSUN2 stimulates SKIL expression in CRC

**FIGURE 3:**
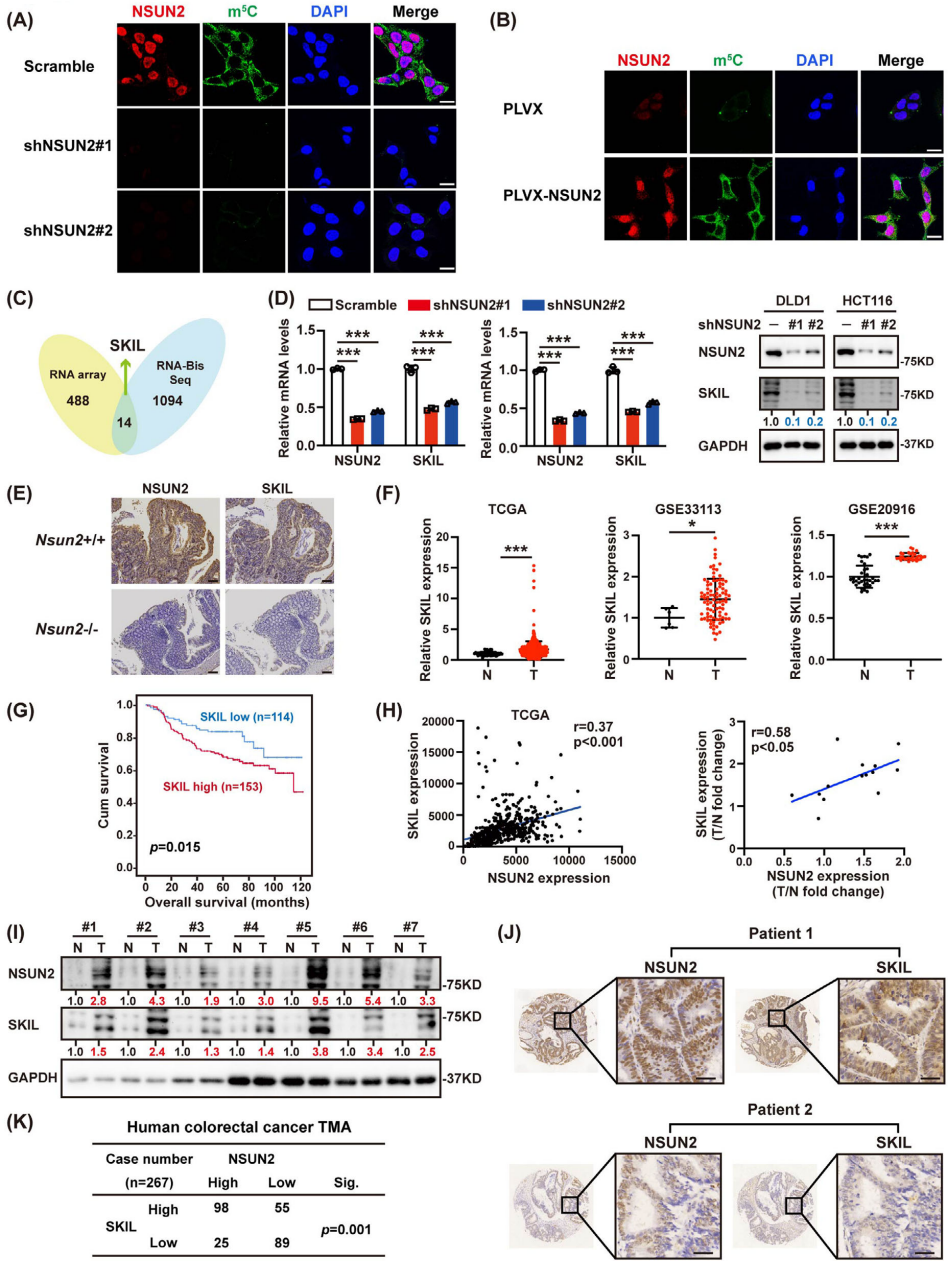
SKIL expression is positively regulated by NSUN2. (A, B) Immunofluorescence staining was performed to detect the NSUN2 and m5C levels with or without NSUN2 knockdown in DLD1 cells (A), or overexpression in HCT116 cells (B). Scale bar = 20 µm. (C) Original data of RNA array was normalized using Affy package in R software, and then the differential gene analysis between samples was carried out and screened by fold‐change and *P* value. Next, the differential genes in RNA array were overlapped with those modified by m5C methylation from Bis‐seq.^30^ (D) SKIL mRNA and protein levels were analyzed after NSUN2 depletion in DLD1 and HCT116 cells. (E) NSUN2 and SKIL immunohistochemistry of colon tumour sections from Nsun2+/+ and Nsun2‐/‐ mice. Scale bar = 100 µm. (F) SKIL was highly expressed in colorectal cancer tumour tissues (T) compared with adjacent normal tissues (N) from TCGA, GSE33113 and GSE20916 databases. (G) Kaplan–Meier estimates of survival time of patients in cohort (*n* = 267) from the sixth Affiliated Hospital of Sun Yat‐sen University by different SKIL expression levels in tumours. (H) NSUN2 were positively correlated with the expression of SKIL at mRNA levels in TCGA and pairs of fresh tissues. (I) Adjacent and tumour tissues of colorectal cancer patients were collected and lysates were analyzed by immunoblotting with indicated antibodies. (J) Representative IHC staining for NSUN2 and SKIL in human CRC tissue microarray (TMA). Case 1 is representative of a patient with NSUN2‐high colon cancer. Case 2 is representative of a patient with NSUN2‐low colon cancer. Scale bar = 100 µm. (K) Quantification of staining intensities of indicated protein from sections in (J). NSUN2 and SKIL show a positive correlation. *P < 0.05; ***P < 0.001.

NSUN2 functions as a methyltransferase that catalyzes the methylation of cytosine to 5‐methylcytosine.^29^ The immunofluorescence assay in NSUN2 knockdown or overexpression CRC cells showed that the m^5^C fluorescence signal was significantly positively correlated with NSUN2 expression (Figure 3A, B), indicating that NSUN2 was responsible for the m^5^C modification level in CRC cells. Therefore, to identify downstream effectors involved in NSUN2‐mediated CRC progression, we assessed potential targets with RNA sequencing and bisulfite sequencing (RNA‐BisSeq) data.^30^ After overlapping, there were 14 potential targets (Figure 3C). Among these, activating transcription factor 3 (*ATF3*),^31^, ^32^ cyclin‐dependent kinase 9 (*CDK9*),^33^ Hes‐related family BHLH transcription factor with YRPW motif 1 (*HEY1*),^34^ Chromobox 4 (*CBX4*),^35^ integrin subunit beta 1 (*ITGB1*),^36^ and SKI like proto‐oncogene (*SKIL*)^27^, ^37^ were oncogenes, while SET domain containing 2 (*SETD2*)^38^ acts as tumour suppressor. Nevertheless, when we used different cell lines for verification, only SKIL expression levels were consistently reduced after NSUN2 knockdown in DLD1 cells, HCT116 cells and HT‐29 cells (Figure 3D and Figure S3A). SKIL, also known as SnoN, is a mediator of the TGF‐β signalling pathway and plays oncogenic or tumour‐suppressive roles in epithelial tissues.^39^ Depletion of NSUN2 significantly reduced SKIL mRNA and protein levels, while NSUN2 overexpression produced the opposite results (Figure 3D and Figure S3B). The SKIL expression level is higher in *Nsun2*+/+ mice than in *Nsun2*‐/‐ mice (Figure 3E). Previous studies revealed that SKIL could promote the tumourigenesis and immune escape of NSCLC cells.^27^ Bioinformatics analysis showed that SKIL was upregulated in CRC (Figure 3F). Analysis of the expression of SKIL in 14 pairs of matched fresh frozen primary CRC tissues and adjacent normal mucosa revealed that CRC exhibited high SKIL levels (Figure S3C). Clinically, patients with high SKIL expression levels were correlated with worse prognosis (Figure 3G). To further assess the clinical relevance between NSUN2 and SKIL, we examined their expression profiles in a cohort of CRC tissues. The results showed that tumour tissues with high NSUN2 expression had higher levels of SKIL (Figure 3H, I). Besides, we performed IHC staining on tissue microarray (TMA). NUSN2 and SKIL showed a significant positive correlation in clinical (Figure 3J, K).

### 2.4 NSUN2 promotes malignant phenotypes of CRC cells via SKIL both in vitro and in vivo

**FIGURE 4:**
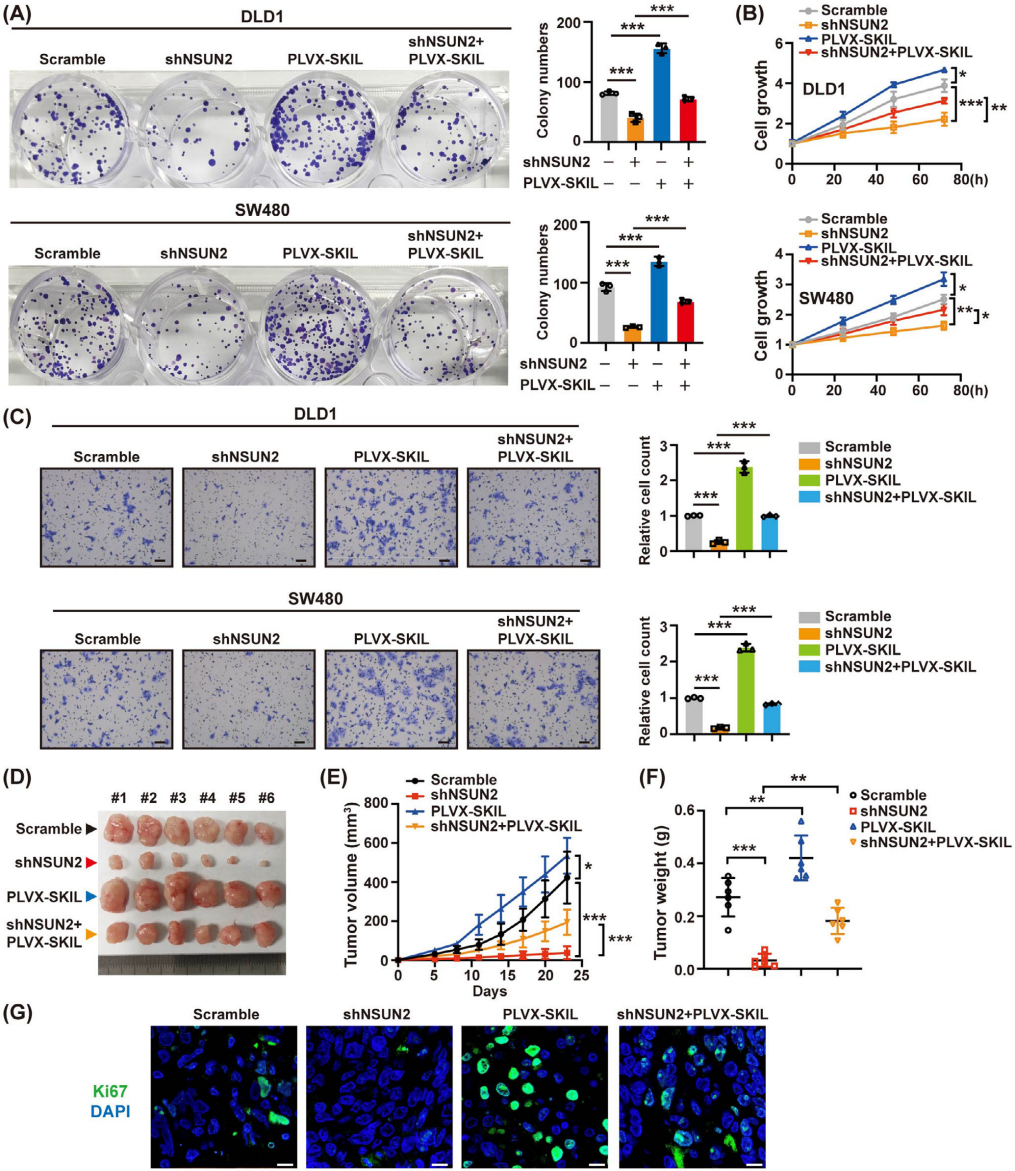
NSUN2‐SKIL axis promotes CRC progression both in vitro and in vivo. (A, B) Colony formation (A), and CCK8 (B) assays were conducted to evaluate the effect of the NSUN2‐SKIL axis on the growth of DLD1 and SW480 cells. (C) Trans‐well assays were conducted to evaluate the effect of the NSUN2‐SKIL axis on the migration of DLD1 and SW480 cells. Scale bar = 100 µm. (D) DLD1 cells were stably infected with the indicated lentivirus and subcutaneously injected into BALB/c‐nude mice (*n* = 6 per group). About 3 weeks after injection, xenografts were removed. Representative images of xenografts were shown. (E, F) Tumour volume (E) and tumour weight (F) were determined. (G) Immunofluorescence was implemented to detect the expression levels of Ki67 using tumour tissues harvested from xenograft model mice. Scale bar = 10 µm. The green colour represents Ki67 staining; the blue colour represents DAPI staining. *P < 0.05; **P < 0.01; ***P < 0.001.

To uncover the function of the NSUN2‐SKIL axis, we then designed rescue functional assays. SKIL overexpression restored the inhibitory effects of NSUN2 knockdown on CRC cell proliferation, colony formation and migration (Figure 4A–C). On the contrary, knockdown of SKIL abrogated the NSUN2 promoting CRC cell colony formation, cell proliferation and migration (Figure S4).

DLD1 cells treated with or without shNSUN2 and PLVX‐SKIL were inoculated into the flanks of nude mice. The results showed that NSUN2 knockdown inhibited tumour growth and decreased tumour weight and Ki67 expression levels, while SKIL overexpression rescued these effects (Figure 4D–G). Altogether, SKIL is a key target of NSUN2 that promotes CRC malignancy.

### 2.5 Methylation by NSUN2 stabilizes SKIL mRNA in a YBX1‐dependent manner

**FIGURE 5:**
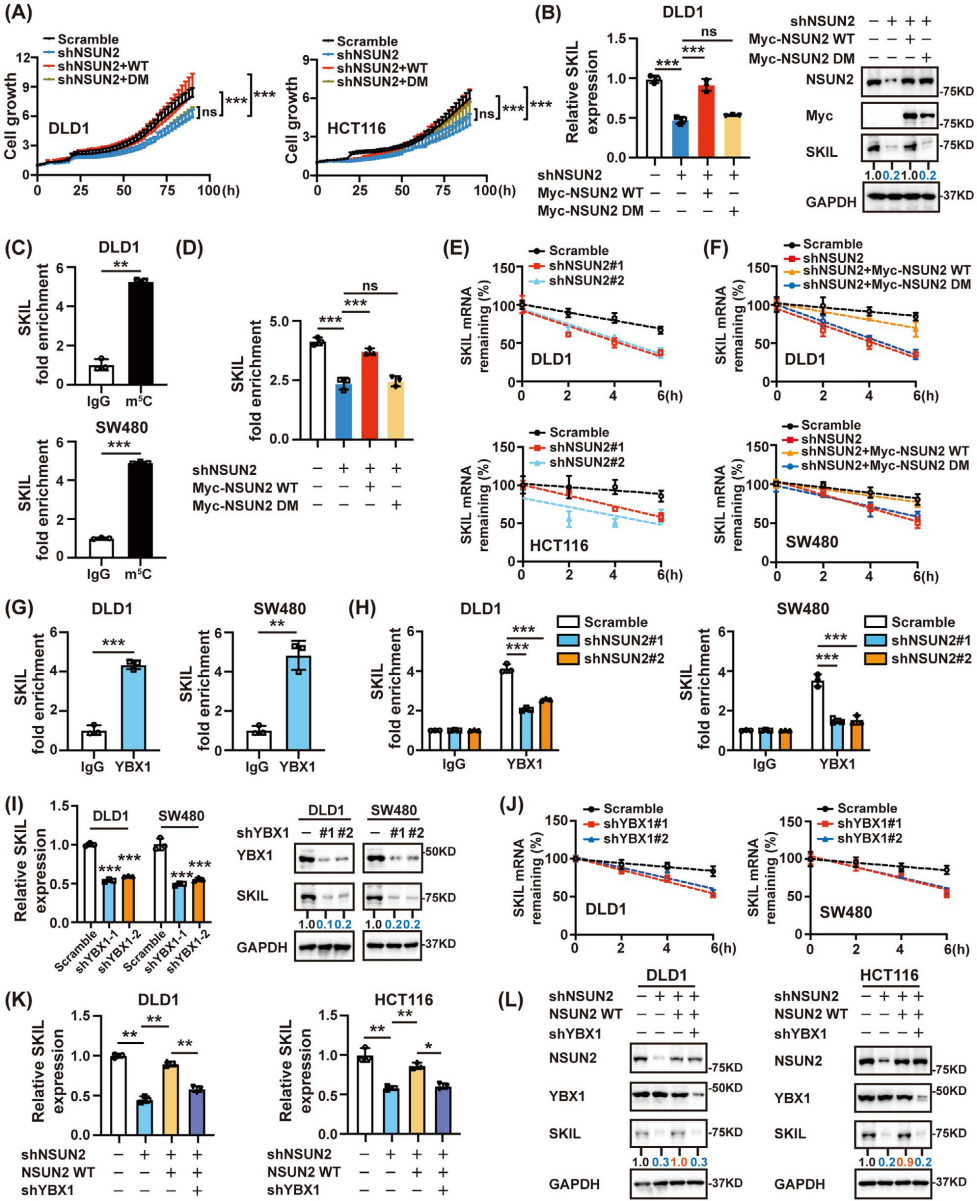
Methylation by NSUN2 stabilizes SKIL mRNA in a YBX1‐dependent manner. (A) Effect of wild‐type or mutant NSUN2 on cell growth in NSUN2 knockdown DLD1 and HCT116 cells by incucyte assays. (B) Wild‐type but not mutant NSUN2 reversed the decrease of SKIL mRNA and protein levels caused by NSUN2 depletion. (C) Assessment of the m5C modification of SKIL mRNA by m5C‐RIP qPCR. (D) Wild‐type but not mutant NSUN2 reversed the decrease of SKIL m5C levels caused by NSUN2 depletion. The “IgG” of each group was normalized as 1. (E) RNA stability assays displayed the reduced SKIL mRNA half‐life in NSUN2 knockdown CRC cells compared with the control cells by qRT‐PCR at the indicated time points after treatment with 10 µg/mL actinomycin D. (F) Wild‐type but not mutant NSUN2 reversed the decrease of SKIL mRNA half‐life induced by NSUN2 silencing. (G) RIP assays showed that YBX1 could bind to SKIL mRNA. (H) Significant reduction of YBX1 binding to SKIL when NSUN2 was silenced. (I) YBX1 knockdown substantially decreased the RNA and protein levels of SKIL. (J) Effects of YBX1 knockdown on mRNA half‐life of SKIL by RNA stability assays. (K, L) Wild‐type NSUN2 reversed the decrease of SKIL mRNA (K) and protein (L) levels caused by NSUN2 depletion, which could be abrogated by YBX1 knockdown. *P < 0.05; **P < 0.01; ***P < 0.001; ns, not significant.

Next, we explored whether the oncogenic function of NSUN2 depends on its m^5^C methyltransferase activity. An enzymatic double‐dead mutant of NSUN2 was generated by introducing point mutations in cysteines 271 and 321. By overexpressing NSUN2 wild‐type (WT) and mutant plasmids in NSUN2‐silenced cells, we found that only the wild‐type was able to rescue CRC cell proliferation and colony formation, while enzymatic dead mutants of NSUN2 failed (Figure 5A and Figure S5A). Additionally, overexpressing WT but not mutant NSUN2 recovered SKIL mRNA and protein levels in NSUN2‐knockdown cells (Figure 5B and Figure S5B). Next, we found that *SKIL* mRNA was effectively enriched by the m^5^C antibody (Figure 5C). Furthermore, silencing NSUN2 decreased the m^5^C levels of SKIL in CRC cells, which could be rescued by overexpressing WT but not mutant NSUN2 (Figure 5D). Since m^5^C modification can regulate target mRNA stability, to determine how NSUN2‐mediated m^5^C regulates the expression of *SKIL* mRNA, we treated CRC cells with actinomycin D. As shown in Figure 5E, NSUN2 silencing significantly reduced the half‐life of *SKIL* mRNA. This reduction could be restored by overexpressing wild‐type but not mutant NSUN2 (Figure 5F), indicating that NSUN2 upregulates SKIL by enhancing its mRNA stability via m^5^C modification.

Y‐box binding protein 1 (YBX1), an important m^5^C reader,^12^ has been proven to maintain mRNA stability by recruiting ELAVL1.^8^ RIP assays using YBX1 antibodies showed that YBX1 could bind to *SKIL* mRNA (Figure 5G), and this interaction was reduced in NSUN2‐silenced cells (Figure 5H), indicating that YBX1 might be the m^5^C reader of SKIL. Further research showed that sh*YBX1* could decrease the expression level of SKIL by reducing the stability of *SKIL* mRNA in CRC cells (Figure 5I, J). Silencing YBX1 suppressed the activation of SKIL expression by NSUN2 (Figure 5K, L). Taking all the above results into consideration, we concluded that NSUN2 could upregulate the expression of SKIL by increasing the stability of *SKIL* mRNA in a YBX1‐dependent manner in CRC cells.

### 2.6 NSUN2 induces the SKIL‐TAZ axis to promote CRC tumourigenesis

**FIGURE 6:**
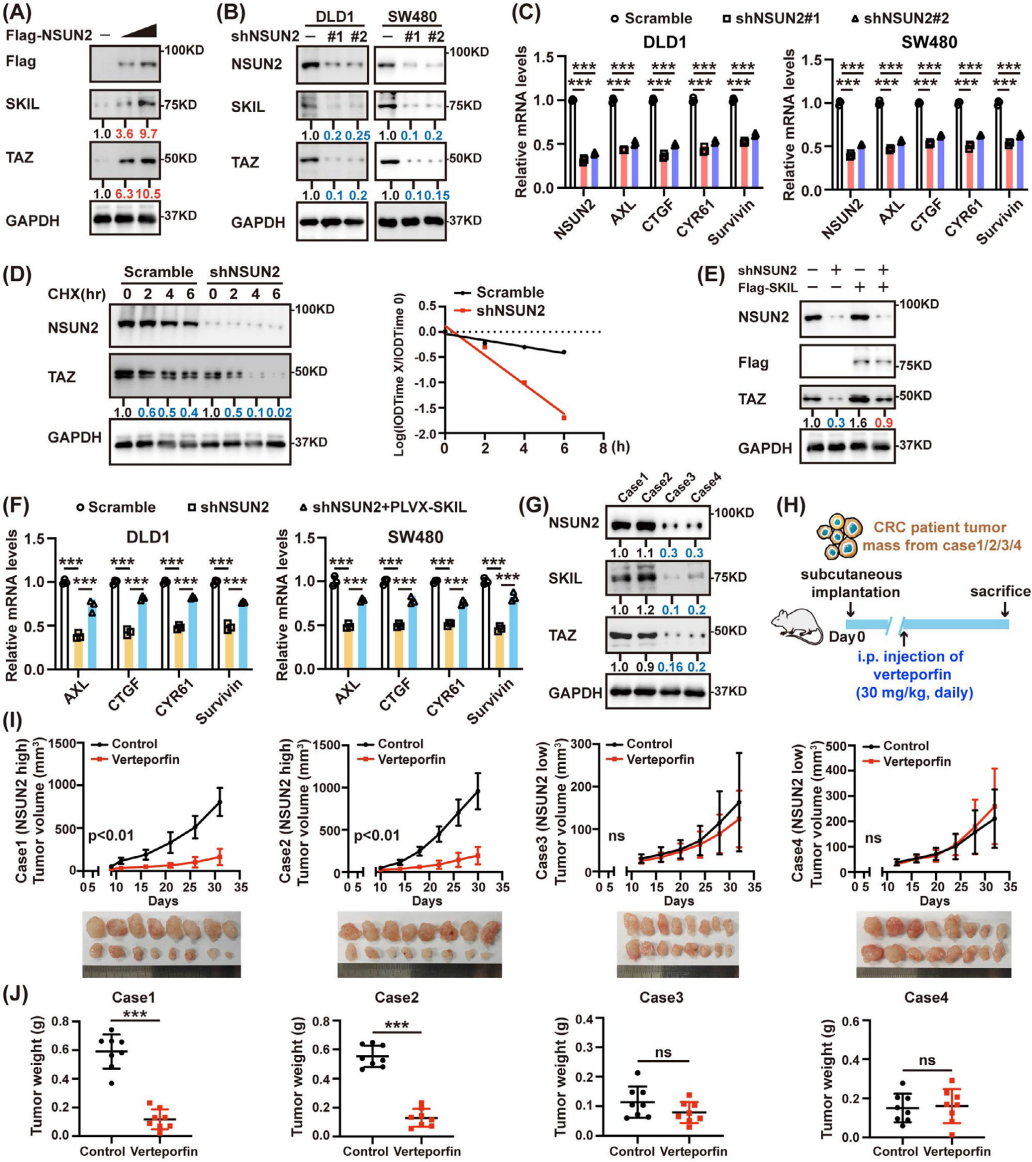
NSUN2 induces the SKIL‐TAZ axis to promote CRC tumourigenesis. (A) Western blot analysis showed that overexpression of NSUN2 led to increased TAZ protein levels. (B) NSUN2 silencing decreased protein levels of TAZ. (C) NSUN2 depletion substantially suppressed the expression of TAZ‐targeted genes. (D) DLD1 cells were infected with shNSUN2 or Scramble and then treated with cycloheximide (100 µg/mL) for indicated times. The turnover of TAZ is indicated graphically. (E) HCT116 cells were transfected with the indicated plasmids and immunoblotted with the indicated antibodies. (F) Overexpression SKIL reversed the inhibition of TAZ‐targeted gene expression caused by NSUN2 silencing. (G) Expression level of NSUN2 in indicated patient‐derived xenografts (PDXs). (H) Treatment schedule of verteporfin is indicated. (I) Impact of verteporfin on tumour growth in mice bearing indicated PDXs (*n *= 8 each group). (J) Impact of verteporfin on tumour weight in mice bearing indicated PDXs (*n* = 8 each group). **P < 0.01; ***P < 0.001; ns, not significant.

YAP/TAZ are closely related transcriptional regulators and participate in the progression of different types of cancer.^40^ SKIL promoted breast cancer tumourigenesis by enhancing the activity of TAZ.^27^ We sought to determine whether NSUN2 affects the SKIL‐TAZ axis. As shown in Figure 6A, B, NSUN2 overexpression increased TAZ protein levels, while NSUN2 knockdown suppressed the TAZ expression. Previous studies have shown that SKIL interacts with multiple components of the Hippo pathway to inhibit the binding of Lats2 to TAZ and the subsequent phosphorylation of TAZ.^26^ Further experiments showed that NSUN2 silencing substantially inhibited TAZ target gene expression in CRC cells (Figure 6C). SKIL silencing accelerated TAZ protein degradation.^27^ Consistently, NSUN2 knockdown increased the TAZ protein turnover rate (Figure 6D). In addition, SKIL overexpression partly restored the activation of TAZ expression and its targets in NSUN2‐depleted cells, indicating that NSUN2 regulates the SKIL‐TAZ axis (Figure 6E, F).

Subsequently, we sought to determine the clinical relevance of NSUN2‐mediated TAZ regulation in a patient‐derived xenograft (PDX) model. To this end, after NSUN2 expression levels were measured (Figure 6G), we implanted fresh primary tumour samples resected from CRC patients into immunocompromised mice and then intraperitoneally injected PBS or verteporfin, a disruptor of YAP/TAZ‐TEAD‐mediated transcription (Figure 6H). Significantly, the administration of verteporfin attenuated the progression of NSUN2‐high PDX tumours (cases 1 and 2). In contrast, verteporfin had a minimal impact on the growth and weight of NSUN2‐low PDX tumours (cases 3 and 4; Figure 6I, J).

**FIGURE 7:**
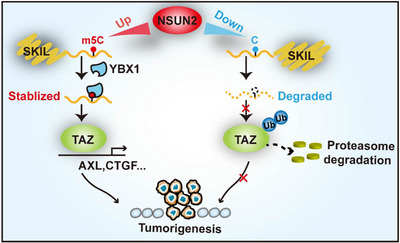
Schematic illustration shows that NSUN2 promotes CRC malignancy by facilitating SKIL mRNA stabilization, followed by TAZ activation.

Collectively, this study revealed that NSUN2 promoted CRC malignancy by facilitating SKIL mRNA stabilization, followed by TAZ activation (Figure 7).

## 3 DISCUSSION

RNA m^5^C modification is an emerging epigenetic modification in recent years that is associated with many cellular and physiological processes (e.g., RNA maturation, editing, localization, stability and translation).^13^, ^41^, ^42^ Abnormal m^5^C modification plays vital roles in various diseases, including cancer^43^, ^44^, ^45^, ^46^ and inflammatory and autoimmune diseases.^47^ Nevertheless, the precise correlations between RNA m^5^C modification and CRC development remain largely unclear. Here, we showed that m^5^C levels were increased in CRC cells due to the overexpression of NSUN2. NOP2/Sun RNA methyltransferases (NSUNs), a notable methyltransferase family, have attracted increasing attention as a result of their functional roles in mRNA modification and the progression of many diseases. NSUN6, a methyltransferase with strong substrate specificity toward mRNA, primarily targets untranslated regions (UTR) at the consensus sequence motif CTCCA. Abnormal expression of NSUN6 is an important factor involved in the regulation of pancreatic cancer cell proliferation.^48^ NSUN3‐dependent RNA modifications drive the translation of mitochondrial mRNA to power metastasis.^41^ In liver cancer, NSUN7 is epigenetically inactivated, which prevents correct mRNA methylation.^49^ m^5^C methyltransferase NSUN2 catalyzes tRNA m^5^C modification, promoting the anaplastic thyroid cancer progression.^19^ NSUN2 activates the ORAI2 expression, promoting gastric cancer metastasis.^50^ We found aberrant overexpression of NSUN2 in CRC tissues, which was highly associated with poor prognosis, suggesting a prognostic value for NSUN2. Our work first reveals the role of NSUN2‐mediated m^5^C modification in the progression of CRC and clarifies its mechanism.

Ski‐like novel gene (SnoN), a member of the Ski family, is an important negative regulator of TGF‐β signalling that was originally identified as a transforming oncogene in chicken embryonic fibroblasts.^23^, ^51^ Both prooncogenic and antioncogenic activities of SnoN have been reported.^28^ In breast cancer, SnoN can antagonize the Hippo kinase complex to promote TAZ signalling, stimulating malignant progression.^26^ SKIL expression has also been found to be higher in NSCLC tissue than in adjacent normal tissue. Silencing of SKIL inhibited the malignant behaviour of NSCLC cells and promoted T‐cell infiltration.^27^ However, in papilloma, overexpression of SnoN inhibits oncogenic transformation induced by Ras and Myc in vitro and significantly blocks tumourigenesis in vivo.^25^ In oesophagal cancer, the role of SnoN in the abrogation of TGF‐β‐induced growth arrest and tumour suppression was explored and confirmed.^52^ Here, we evaluated the expression levels of SKIL in different CRC databases and uncovered that SKIL was highly expressed in CRC patients. In addition, SKIL expression was assessed in CRC tissues compared with paired normal tissues. Kaplan–Meier analysis revealed that patients with high SKIL levels had shorter overall survival times. Altogether, our results demonstrated the oncogenic role of SKIL in CRC and its potential as a prognostic indicator for CRC patients. Until now, the mechanisms controlling the expression of SKIL have remained unclear. Our findings revealed a new mechanism of SKIL expression regulation through m^5^C modification, which suggests that the regulatory mechanism of SKIL expression is epigenetically mediated and that the inhibition of m^5^C modification in SKIL may have therapeutic benefits for CRC patients. In vitro and in vivo experiments demonstrate that NSUN2 promotes CRC development through SKIL. In CRC tumour tissues, the protein expression levels of NSUN2 and SKIL showed a significant positive correlation. Therefore, it may be interesting to further study the synergistic anticancer effect of SKIL and NSUN2‐mediated m^5^C modification in CRC.

Recently, an integrated m^5^C regulatory network has been determined. The regulatory effects of RNA modification are usually interpreted by reader proteins.^8^ Aly/REF export factor (ALYREF), characterized as the m^5^C “reader” in the nucleus, could facilitate the export of m^5^C‐modified mRNAs.^53^ Upon NSUN2 deficiency, the recognition of *CDKN1A* mRNA by ALYREF was suppressed, resulting in a decrease in *CDKN1A* mRNA shuttling from the nucleus to the cytoplasm.^54^ In addition, YBX1 could recognize m^5^C‐modified mRNAs through the indole ring of W65 in its cold‐shock domain. YBX1 maintains the stability of its target mRNA by recruiting ELAVL1.^8^ In this study, we uncovered m^5^C‐mediated RNA stabilization of SKIL by NSUN2, which was YBX1 dependent. Whether any other m^5^C mediators are involved in SKIL regulation remains to be elucidated. Moreover, recent studies have revealed that m^5^C RNA modifications of tRNA play important roles in different biological functions.^45^ For instance, the oxidative phosphorylation complex subunits translation depends on the formation of m^5^C in mitochondrial tRNA^Met^ by NSUN3. Neuronal Nsun2‐deficiency decreased the m^5^C levels of tRNA, resulting in inhibited expression of 70% of tRNA^Gly^ isodecoders.^55^ Therefore, the role of m^5^C‐mediated tRNA methylation in CRC tumourigenesis needs to be further investigated. Furthermore, it has been reported that the P53 pathway, Wnt/β catenin signalling and PI3K/AKT/mTOR signalling were significantly correlated with m^5^C levels.^56^ Given the important roles of these pathways in CRC, whether m^5^C modification impacts these pathways in CRC needs further exploration.

TAZ is the key transducer of Hippo signalling and a novel downstream signalling transducer of PODXL in mediating cancer invasiveness and stemness properties in colon cancer.^57^ Here, we showed that NSUN2 post‐transcriptionally stabilized SKIL, triggering the abnormal activation of TAZ. The benzoporphyrin derivative, verteporfin is a powerful photosensitizer and widely used in the treatment of macular degeneration. Within the syngeneic tumour microenvironment, verteporfin could inhibit the expression of PD‐L1, which was associated with enhanced T‐lymphocyte infiltration.^58^ Previous study found that verteporfin preferentially induced apoptosis of cultured patient‐derived EGFR‐amplified/mutant Glioblastoma (GBM) cells, suppressed expression of YAP/TAZ transcriptional targets and conferred significant survival benefit in an orthotopic xenograft GBM model.^59^ Additionally, the researchers further designed and initiated a phase 0 clinical trial of Visudyne, an FDA‐approved liposomal formulation of verteporfin. As TAZ is the main target of verteporfin,^60^ we explored the associations between NSUN2 expression levels and verteporfin based on PDX models. Results indicated that tumours with high NSUN2 expression are more sensitive to verteporfin treatment, hinting at its potential therapeutic effects in CRC.

Our study has some limitations. First, *Nsun2*‐/‐ mice were whole body knock out. The systemic knockout may exert effect on different cell types. Intestinal conditional knockout (*Nsun2* ^CKO^) mice would be better to further explore the role of NSUN2 in CRC progression. Additionally, 5‐fluorouracil (5‐FU) is the first‐line treatment for colorectal cancer.^61^ High NSUN2 expression lowers the sensitivity of fluorouracil in prostate cancer.^62^ Further research on combinatorial effects of verteporfin and 5‐FU may help to improve therapeutic efficacy in CRC.

## 4 MATERIALS AND METHODS

### 4.1 Cell culture and transfection

The human CRC cell lines DLD1, SW480, HCT116, RKO and HT‐29 were purchased from the American Type Culture Collection (ATCC). The cells were cultured in RPMI‐1640 (Roswell Park Memorial Institute) or Minimum Essential Medium medium supplemented with 10% fetal bovine serum (Gibco) at 37°C, 5%CO_2_. All cell lines are routinely tested for mycoplasma.

The cDNAs for human NSUN2 and human SKIL were cloned by RT‐PCR and subcloned into pcDNA3.1 (+) vector or PLVX vector. The double mutants of NSUN2 (C271A, C321A) were constructed using Mut Express II Fast Mutagenesis Kit v2 (C214‐02, Vazyme). The primers were listed as follows: NSUN2 (C321A) F: GTATTCCACGgcTTCACTAAACCCTATTGAGGATGAAG; R: AGTGAAgcCGTGGAATACACCATCCTTCCACCT. NSUN2 (C271A) F: TATGTGATGTCCCTgcCAGTGGAGACGGCACTATGAGA; R: TGgcAGGGACATCACATAAAATTCGATCATAGA. The target plasmid was amplified using Phanta Max Super‐Fidelity DNA Polymerase. Then, Dpn I digestion was performed to remove the methylated template plasmid. The amplified digestive product recombined the mutant site under the catalysis of Exnase II, achieving in vitro cyclization of linear DNA. Finally, the mutant plasmids were sequenced by BGI Genomics. Transient transfection was performed using polyethyleneimine HCl (polyysciences Inc,24765‐1) according to the manufacturer's instructions.

### 4.2 Animals

NU/NU nude mice (#D000521) and C57BL/6 mice (#N000013) were purchased from GemPharmatech Biotechnology Corporation. All mice were crossed at least five generations to a C57BL/6 background. Littermates were used as control for experiments. Female nude mice, aged 4 weeks, were utilized for the xenograft studies. They were randomly assigned to various groups. For the PDX, PDX models were obtained from the Sixth Affiliated Hospital of Sun Yat‐sen University. All mice were maintained under pathogen‐free conditions. Animal experiments were approved by the Institutional Animal Care and Use Committee of The Sixth Affiliated Hospital of Sun Yat‐sen University (IACUC‐2022101101).

### 4.3 AOM‐DSS model

*Nsun2* whole body knock‐out mice (*Nsun2*‐/‐) were purchased from GemPharmatech Biotechnology Corporation (Strain no. T013748). The AOM (10 mg/kg body weight) was administered to the mice via intraperitoneal injection on the first day. Five days later, mice were fed with 2% DSS in drinking water for 6 days and were then followed by 2 weeks of recovery. The 2% DSS treatment‐2 weeks recovery cycle were repeated once and at last followed by a 1.5% DSS treatment for 3 days. Mice were sacrificed at 80 days after AOM injection.

### 4.4 Xenograft tumour model in nude mice

For xenograft studies, female BALB/c nude mice (4 weeks old) were used and randomly divided into three groups. DLD1 cells infected with shNSUN2, PLVX‐NSUN2 or control were harvested and injected into the flanks of mice. Besides, to study the function of the NSUN2‐SKIL axis in CRC tumourigenesis, nude mice were randomly divided into four groups (Scramble, shNSUN2, PLVX‐SKIL, shNSUN2+PLVX‐SKIL). Cells were resuspended in PBS at a density of 1.5 × 10^7^ cells/mL and injected into the flanks of the nude mice. Subsequently, randomization and single blinding were performed when measuring tumour volume which was calculated as length×width^2^×0.5. About 2 weeks later, the tumours were carefully removed, photographed and weighed.

### 4.5 PDX models

Patient‐derived tumour fragments were injected into the flanks of female NCG (NOD/ShiLtJGpt‐*Prkdc^em26Cd52^Il2rg* ^em26Cd22/^Gpt) mice (GemPharmatech Biotechnology Corporation). When the tumour sizes reached about 200 mm^3^, the mice were randomly divided into two groups and subjected to the treatment of PBS and verteporfin 30 mg/kg daily (intraperitoneal injection), respectively. The tumour volumes were measured and calculated as length×width^2^×0.5. Finally, the tumours were harvested at the end of the experiments.

### 4.6 Immunoblotting

Cells were harvested with a lysis buffer containing a proteinase and phosphatase inhibitors cocktail. Then cell lysates were subjected to SDS‐PAGE. After transferring, blocking and incubating with specific antibodies, the proteins on the membranes were visualized. The antibodies used included NSUN2 (1:4000, Proteintech, 20854‐1‐AP), SKIL (1:4000, Proteintech, 19218‐1‐AP), GAPDH (1:5000, Proteintech, 60004‐1‐Ig), Myc (1:5000, CST, 2276s), Flag (1:5000, CST, 8146s), TAZ (1:4000, Proteintech, 22306‐1‐AP) and YBX1 (1:4000, abcam, ab12148).

### 4.7 Immunohistochemistry

Briefly, sections were de‐paraffinized, re‐hydrated, retrieved antigen and incubated with anti‐NSUN2, anti‐SKIL or anti‐Ki67 at 4°C overnight. Next, the slides were incubated with an HRP‐conjugated secondary antibody, visualized with DAB (Dako, Cytomation) and counterstained with hematoxylin. Image J was used for quantification.

### 4.8 Immunofluorescence

Cells were washed with PBS and fixed with 4% paraformaldehyde for 10 min at room temperature. Then cells were permeabilized with 0.5% Triton X‐100 for 20 min at room temperature, blocked and incubated with anti‐NSUN2 (1:200, Proteintech, 20854‐1‐AP), anti‐m^5^C (1:100, abcam, ab10805), or anti‐Ki67 (1:100, CST, 9449) at 4°C overnight. Afterwards, cells were washed and incubated with fluorochrome‐conjugated secondary antibody (1:500) for 1 h at room temperature followed by DAPI (Beyotime, C1002) staining. Images were acquired with confocal microscopy.

### 4.9 RNA isolation and real‐time qPCR

Total RNA was isolated from cells with TRIZOL reagent (Invitrogen). The cDNA was prepared using ReverTra Ace qPCR RT Master Mix with gDNA Remover (TOYOBO). Then the gene expression was quantified using 2×SYBR Green qPCR Master Mix (biotool, #B21203) in the LightCycler480 PCR system (Roche). Finally, the results were analyzed by the 2(^−ΔΔCT^) method and GAPDH was used as an internal control. All of the qPCR primers are listed in Table S2.

### 4.10 RNA array

DLD1 cells were infected with scramble or shNSUN2 lentivirus for 48 h. Then RNA samples from DLD1 cells were isolated using the Trizol reagent (Invitrogen) and sent to Shanghai Biotechnology Corporation for sequencing based on Affymetrix Human U133 Plus 2.0 Array.

### 4.11 Cell proliferation and colony formation assays

The incucyte live cell analysis system (Essen Bioscience) is used for cell proliferation assay. Cells were seeded in six‐well plates and placed inside incubators, then the machine would automatically capture amounts of images at different time points and analyze them while cells remained unperturbed. For colony formation assay, 800–1000 infected CRC cells were seeded in six‐well plates and cultured in indicated mediums for 10 days. Then, cells were fixed with 4% paraformaldehyde and stained with a crystal violet solution. The numbers of colonies were counted (*n* = 3).

### 4.12 Cell migration assay

1×10^5^ infected cells were placed in the upper chamber and incubated with serum‐free medium. Simultaneously, culture medium containing 10% FBS was added to the basolateral chamber. Twenty‐four hours later, cells were fixed with 4% paraformaldehyde for 10 min, washed with PBS and then stained with 0.1% crystal violet for 10 min. Finally, the cells were photographed in three random visual fields and analyzed by image J.

### 4.13 m5C methylated RNA immunoprecipitation

The total RNA of cells was extracted with TRIZOL reagent (Invitrogen), then fragmented into ～200 nucleotides (nt) by incubation with fragmentation buffer (Ambion) at 70°C for 10 min. Ten percent volume RNA was used as input, while the remaining RNA was incubated with anti‐m^5^C antibody, followed by incubated with protein A/G Magnetic Beads in 900 mL of RIP lysis buffer on a rotary homogenizer at 4°C for 4 h. Next, beads were resuspended with a mixture of 10 mL of 10% SDS, 10 mL of Proteinase K and 130 mL of RIP buffer and digested at 55°C for 30 min. RNA in immunoprecipitation or input group was recovered according to the manufacturer's instruction.

### 4.14 mRNA stability assay

DLD1, HCT116 and SW480 cells were infected with the indicated virus and then incubated with actinomycin D (10 µg/mL) for indicated time points (0, 2, 4, or 6 h). Finally, RNA was isolated with Trizol and quantified with qPCR.

### 4.15 RNA immunoprecipitation

After preparing and collecting cells, RNA immunoprecipitation was conducted as described previously,^61^ by the Magna RIP RNA‐binding protein immunoprecipitation kit (17‐701, Millipore). In brief, cells were lysed on ice with RIP lysis buffer supplemented with a 0.1% RNase inhibitor and 0.2% protease inhibitor cocktail and then subjected to immunoprecipitation. The immunoprecipitated complex was treated with Proteinase K. Finally, the RNA was purified, collected and converted to cDNA for qPCR.

### 4.16 TMA analysis

For TMA, the original immunohistochemistry slides were scanned by Aperio Versa (Leica Biosystems) which captured digital images of the immunostained slides. The receiver operating characteristic curve was used to define the cutoff point when calculating the survival rate.

### 4.17 Bioinformatics analysis

Colorectal cancer data sets were downloaded from the publicly available GEO databases (https://www.ncbi.nlm.nih.gov/gds/?term = GSE), including GSE20916, GSE33113, GSE8671 and GSE17538. Bioinformatics analysis and visualization were performed by SPSS and GraphPad software, version 8.

### 4.18 Statistical analysis

Survival analysis and Kaplan–Meier analyses were done by SPSS software. The *P* values were calculated by log‐rank test. Statistical analysis was performed using GraphPad software, version 8. Differences between the two groups or multiple groups were calculated by unpaired Student's *t‐*tests or one‐way analysis, respectively. The data are presented as the mean ± standard deviation (SD). *P* value <0.05 was considered significant.

## AUTHOR CONTRIBUTIONS

Lekun Fang conceived and designed the study. Shaomin Zou, Yizhi Huang, Ziqing Yang, Jieping Zhang, Manqi Meng, Yijing Zhang, Rui Sun, Weiyao Li, Jesús García‐Foncillas López and Lekun Fang performed the experiments. Junyan Feng and Wencong Wang performed the animal breeding. Shaomin Zou wrote the manuscript with the assistance of Lekun Fang. Shaomin Zou and Lekun Fang analyzed the TMA results. All authors reviewed the manuscript.

## CONFLICT OF INTEREST STATEMENT

The authors declare no conflict of interest.

## ETHICS APPROVAL AND CONSENT TO PARTICIPATE

All cancer patient samples were collected with the patients’ written informed consent and approval from the Institutional Review Board of the Sixth Affiliated Hospital of Sun Yat‐sen University (2023ZSLYEC‐366). Animal experiments were approved by the Institutional Animal Care and Use Committee of The Sixth Affiliated Hospital of Sun Yat‐sen University (IACUC‐2022101101).

## Supporting information

Supporting Information

## Data Availability

All data generated or analysed during this study are included in the article and are available from the corresponding author upon reasonable request.
