# Supplementary material for: NSUN2 promotes colorectal cancer progression by enhancing SKIL mRNA stabilization
Source: Clin Transl Med. 2024 Mar 11;14(3):e1621. doi: 10.1002/ctm2.1621 (PMC10928349; doi:10.1002/ctm2.1621)
Supplement: Supplementary file 1 — Supporting Information [file CTM2-14-e1621-s001.docx]

**Supporting information**

**NSUN2 promotes colorectal cancer progression by enhancing SKIL mRNA stabilization**

Shaomin Zou^1,2,3†^ | Yizhi Huang^1,4†^ | Ziqing Yang^1,2,3^ | Jieping Zhang^1,2,3^ | Manqi Meng^1,2,3^ | Yijing Zhang^1,2,3^ | Junyan Feng^1,2,3^ | Rui Sun^1,2,3^ | Weiyao Li^1,2,3^ | Wencong Wang^1,2,3^ | Jesús García-Foncillas López^5^ | Lekun Fang^1,2,3^

This file contains:

- Supplementary Figure S1-S5

- Supplementary Table S1-S2


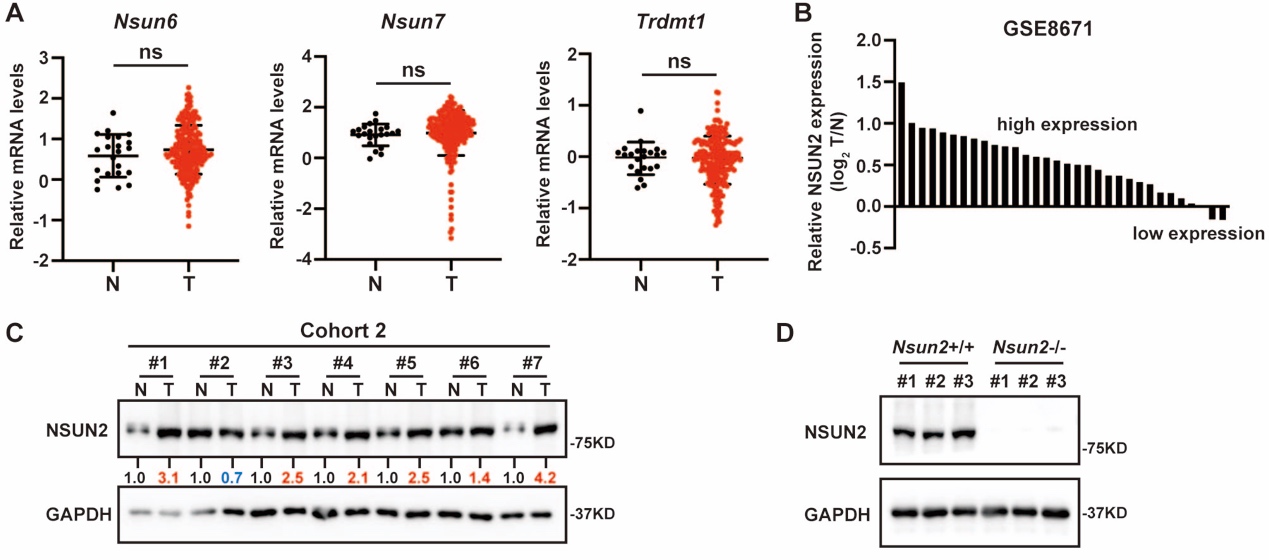


**Supplementary Fig.S1** NSUN2 is high expressed in CRC. (A) qT-PCR analysis showed the NSUN6, NSUN7 and TRDMT1 mRNA levels in colorectal cancer tumor tissues from TCGA. (B) NSUN2 was highly expressed in colorectal cancer tumor tissues (T) compared to adjacent normal tissues (N) from GSE8671 databases. (C) Western blot analysis showed the NSUN2 protein levels in paired samples of CRC tumors (T) and corresponding adjacent normal tissues (N) in cohort 2. (D) NSUN2 expression in colon tissues from *Nsun2*+/+ and *Nsun2*-/- mice were determined by western blot.


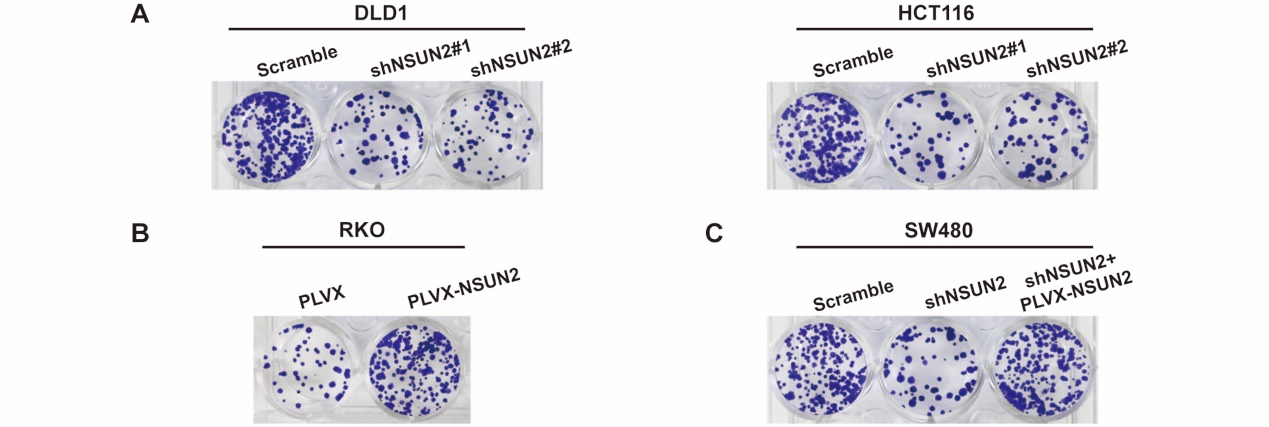


**Supplementary Fig.S2** NSUN2 promotes colony formation of CRC cells. (A) Effects of NSUN2 depletion on the clonogenic ability of DLD1 and HCT116 cells. (B) Overexpression NSUN2 promoted CRC cell colony formation. (C) Wild-type NSUN2 restored the reduced colony numbers caused by NSUN2 silencing.


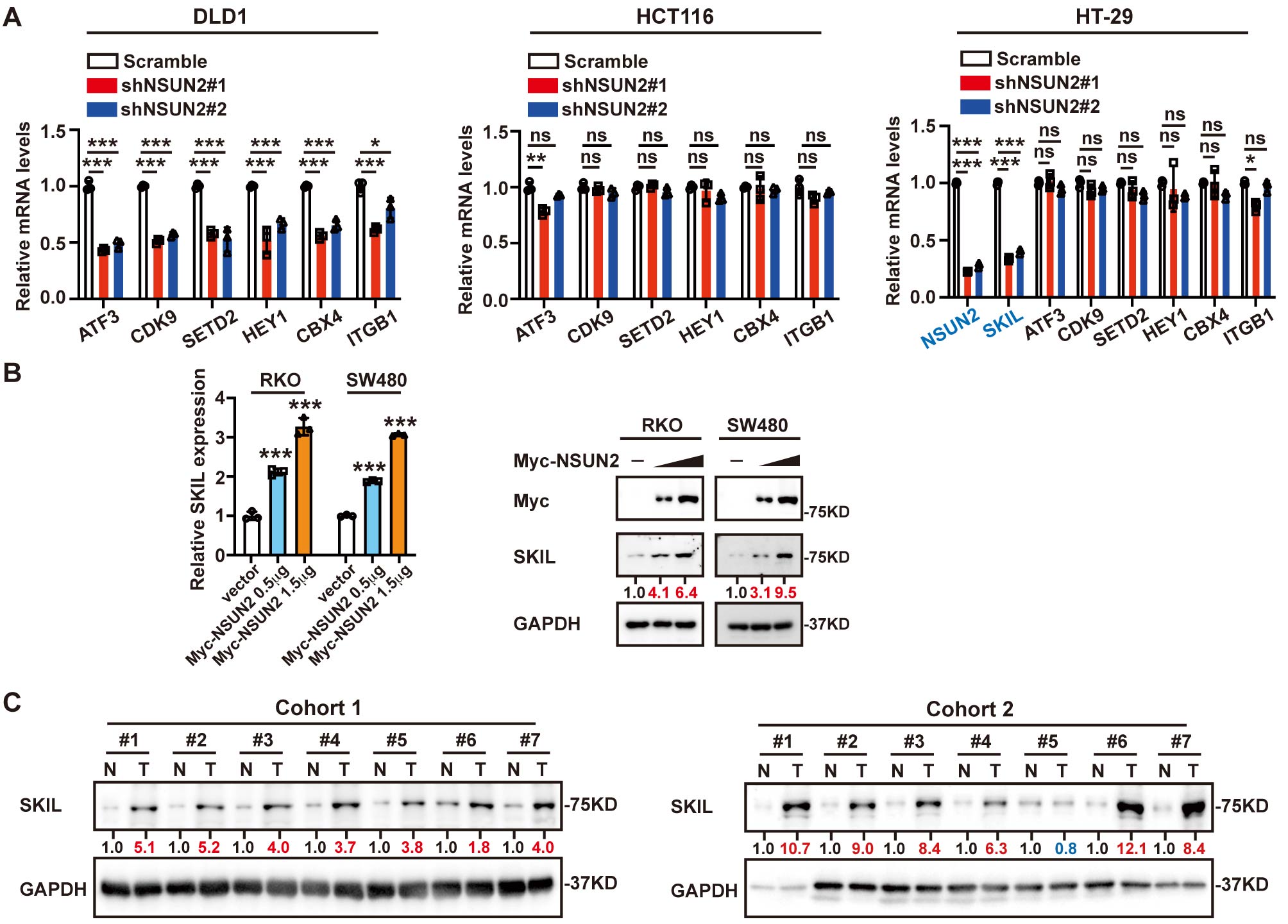


**Supplementary Fig.S3** NSUN2 activates SKIL expression. (A) NSUN2, SKIL, ATF3, CDK9, SETD2, HEY1, CBX4 and ITGB1 mRNA levels were analyzed after NSUN2 depletion. (B) Overexpression of NSUN2 increased the expression of SKIL in RKO and SW480 cells. (C) Western blot analysis showed the SKIL protein levels in paired samples of CRC tumors (T) and corresponding adjacent normal tissues (N).


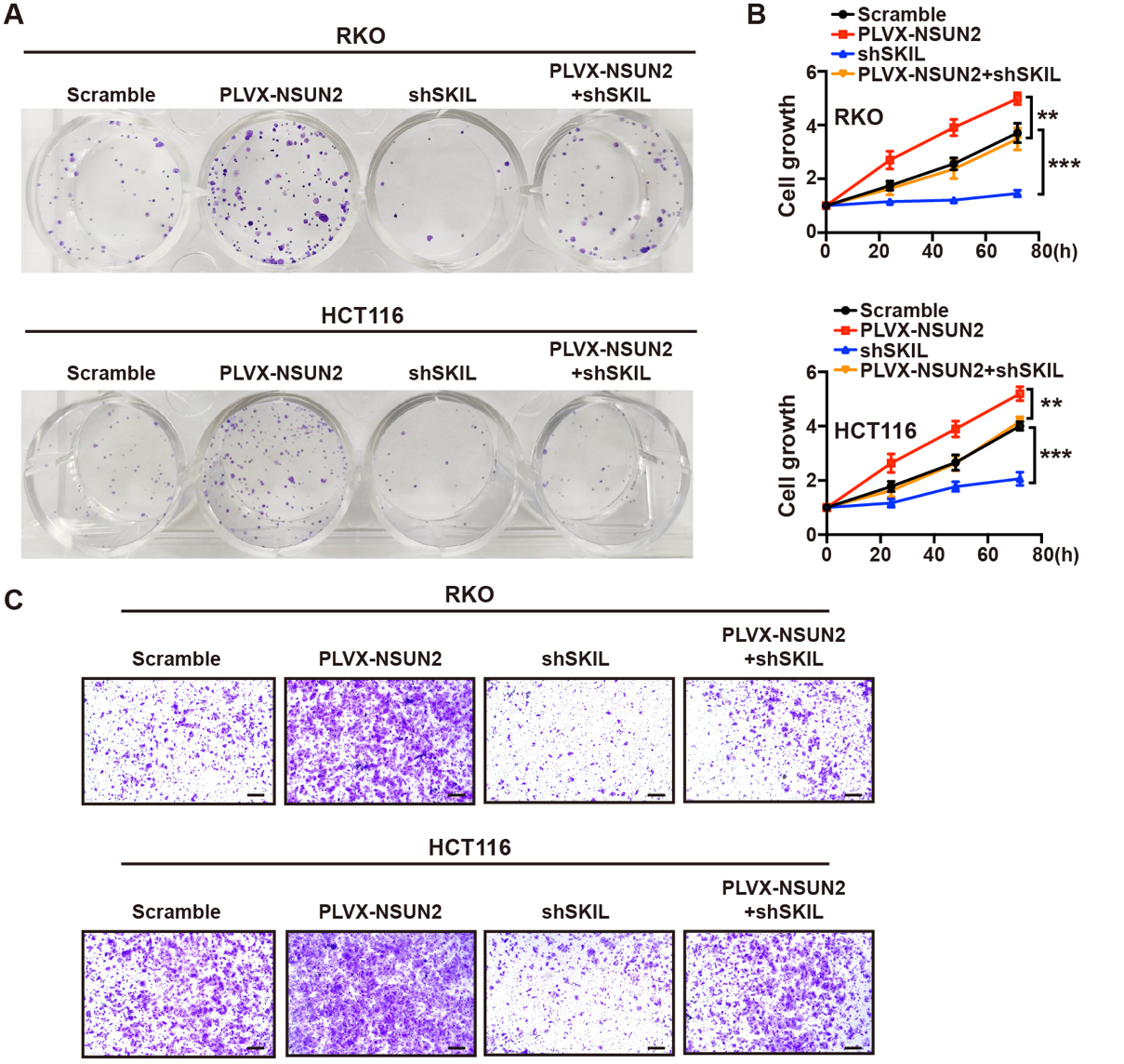


**Supplementary Fig.S4** NSUN2-SKIL axis promotes CRC progression *in vitro.* (A) Colony formation (A), and CCK8 (B) assays were conducted to evaluate the effect of NSUN2-SKIL axis on the growth of RKO and HCT116 cells. (C) Trans-well assays were conducted to evaluate the effect of NSUN2-SKIL axis on the migration of RKO and HCT116 cells. Scale bar: 100μm.


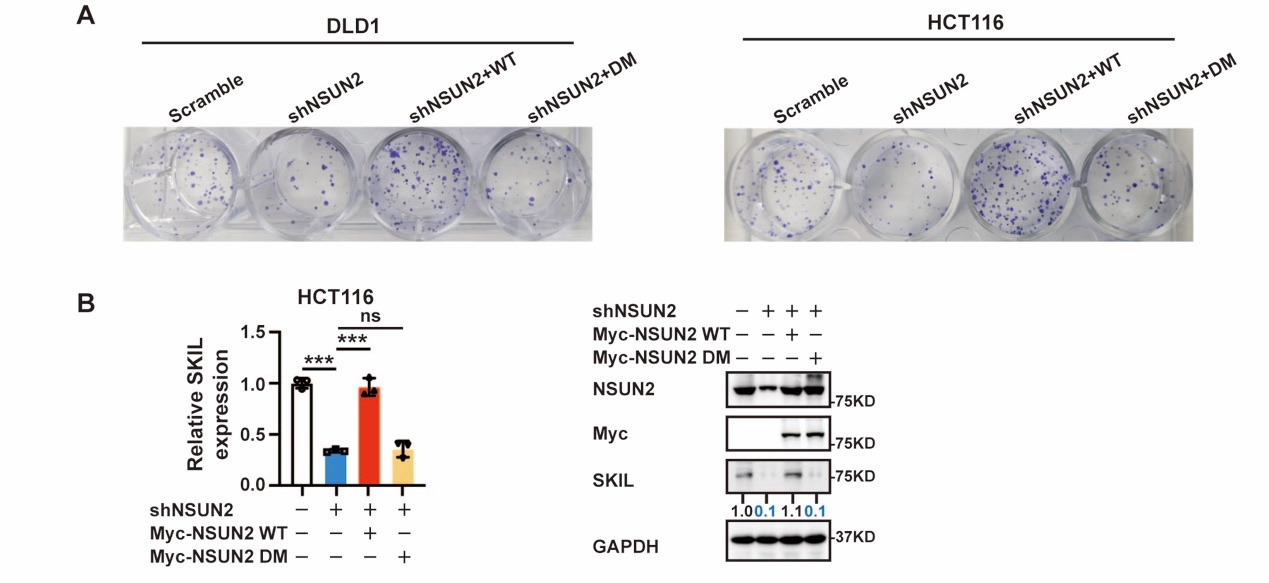


**Supplementary Fig.S****5** NSUN2 activates SKIL expression dependent on its m^5^C methyltransferase activity. (A) Effect of wild-type or mutant NSUN2 on cell growth in NSUN2 knockdown CRC cells by colony formation assays. (B) Wild-type but not mutant NSUN2 reversed the decrease of SKIL mRNA and protein levels caused by NSUN2 depletion.

**Supplementary Table S1. Correlation between NSUN2 expression and clinicopathological features of colorectal cancer patients.**

|  | NSUN2 expression | | |
| --- | --- | --- | --- |
| Variable | Low | High | p value^a^ |
| Gender |  |  | 0.6 |
| Male | 76 | 61 |  |
| Female | 68 | 62 |  |
| age |  |  | 0.06 |
| <59 years | 75 | 50 |  |
| ≥59 years | 69 | 73 |  |
| Histological grade |  |  | 0.07 |
| G1 | 12 | 8 |  |
| G2 | 103 | 102 |  |
| G3 | 29 | 13 |  |
| pT status |  |  | 0.77 |
| T1 | 5 | 4 |  |
| T2 | 16 | 19 |  |
| T3 | 121 | 98 |  |
| T4 | 2 | 2 |  |
| pN status |  |  | 0.86 |
| N0 | 91 | 79 |  |
| N1 | 53 | 44 |  |
| pM status |  |  | 0.07 |
| M0 | 128 | 109 |  |
| M1 | 16 | 14 |  |
| Clinical stage |  |  | 0.44 |
| I | 13 | 19 |  |
| II | 67 | 52 |  |
| III | 48 | 38 |  |
| IV | 16 | 14 |  |

**Supplementary Table S2. Primers for qPCR**

| Gene | Forward primer sequence | Reverse primer sequence |
| --- | --- | --- |
| *Nsun2* | CAAGCTGTTCGAGCACTACTAC | CTCCCTGAGAGCGTCCATGA |
| *Skil* | GTTAAGCGAACCTGTACTTCTGT | GTAGGCGACATGCTTTCTTGG |
| *Atf3* | CCTCTGCGCTGGAATCAGTC | TTCTTTCTCGTCGCCTCTTTTT |
| *Cdk9* | ATGGCAAAGCAGTACGACTCG | GCAAGGCTGTAATGGGGAAC |
| *Setd2* | TGCTTCTAGTCGATTTTTGCCC | AGGGTTTGGAGTATCACTTTGC |
| *Hey1* | GTTCGGCTCTAGGTTCCATGT | CGTCGGCGCTTCTCAATTATTC |
| *Cbx4* | GCAGAGTGGAGTATCTGGTGA | AGCTTGGCACGGTTGTCAG |
| *Itgb1* | CCTACTTCTGCACGATGTGATG | CCTTTGCTACGGTTGGTTACATT |
| *Ybx1* | GGGGACAAGAAGGTCATCGC | CGAAGGTACTTCCTGGGGTTA |
| *Axl* | GTGGGCAACCCAGGGAATATC | GTACTGTCCCGTGTCGGAAAG |
| *Ctgf* | CAGCATGGACGTTCGTCTG | AACCACGGTTTGGTCCTTGG |
| *Cyr61* | CTCGCCTTAGTCGTCACCC | CGCCGAAGTTGCATTCCAG |
